# Genetic Manipulation of Non-tuberculosis Mycobacteria

**DOI:** 10.3389/fmicb.2021.633510

**Published:** 2021-02-17

**Authors:** Nyaradzai Mitchell Chimukuche, Monique J. Williams

**Affiliations:** Department of Molecular and Cell Biology, University of Cape Town, Cape Town, South Africa

**Keywords:** non-tuberculosis mycobacteria (NTMs), mutagenesis, homologous recombination, recombineering, transposon mutagenesis, CRISPR/Cas

## Abstract

Non-tuberculosis mycobacteria (NTMs) comprise a large group of organisms that are phenotypically diverse. Analysis of the growing number of completed NTM genomes has revealed both significant intra-genus genetic diversity, and a high percentage of predicted genes that appear to be unique to this group. Most NTMs have not been studied, however, the rise in NTM infections in several countries has prompted increasing interest in these organisms. Mycobacterial research has recently benefitted from the development of new genetic tools and a growing number of studies describing the genetic manipulation of NTMs have now been reported. In this review, we discuss the use of both site-specific and random mutagenesis tools in NTMs, highlighting the challenges that exist in applying these techniques to this diverse group of organisms.

## Introduction

The genus *Mycobacterium* is a diverse group of organisms that includes more than 180 species. Species within the genus that do not cause tuberculosis (*Mycobacterium tuberculosis* complex) or leprosy (*Mycobacterium leprae* and *Mycobacterium lepromatosis*) are termed non-tuberculosis mycobacteria (NTMs) or environmental mycobacteria. While most of these species are non-pathogenic and occur ubiquitously in the environment, several are opportunistic pathogens causing infection of the lungs, soft tissue, and bones (Griffith et al., 2007).

Mycobacterial research has benefitted from two major advances in the last two decades. Firstly, improvements in next-generation sequencing technology has rapidly increased the number of completed whole genome sequences; currently more than 150 mycobacterial genomes are publicly available (Gupta et al., 2018). Secondly, the development of new genetic tools, particularly those for efficiently generating site-specific mutations in mycobacteria, has facilitated the study of gene function. Although *in silico* tools can provide some insight into gene function, these predictions require experimental validation (Niehaus et al., 2015) and rely on inferences from homologs. Analysis of 41 NTM genomes revealed that 61% of the predicted genes could not be assigned a function by genome annotation tools (Fedrizzi et al., 2017), highlighting the need for functional studies within NTMs. One of the major advantages of using site-specific mutagenesis to elucidate gene function is the ability to study gene products in their cellular context. In the case of clinically relevant NTMs, this is crucial for understanding NTM pathogenesis and disease (Russell et al., 2010), and for the development of more effect antibiotics to treat NTM infections (Wu et al., 2018).

Studying NTM physiology and gene function also has relevance for other diseases. Heat-killed *Mycolicibacterium vaccae* (SRL 172) has shown some potential in the treatment of prostate (Hrouda et al., 1998) and lung cancer (O’Brien et al., 2000), suggesting that NTMs could have utility as immunomodulators. This is consistent with the hypothesis that NTM-exposure influences BCG vaccine efficacy in preventing pulmonary tuberculosis in different populations (Fine, 1995). Apart from impacting human health, NTMs also cause disease in animals, and infection of ruminants by *Mycobacterium avium* ssp. *paratuberculosis* results in substantial losses for the livestock industry (Whittington et al., 2019). NTMs may also be useful for bioproduction; *Mycolicibacterium smegmatis* has been used as an alternative to *Escherichia coli* for recombinant protein production (Goldstone et al., 2008) while the large number of genes unique to mycobacteria represent an untapped resource for novel bio-transformations and small molecule production. The genetic manipulation of NTMs therefore has significance for several areas.

Herein, we review studies that have undertaken genetic manipulation in NTMs, discussing the advantages and disadvantages of each mutagenesis method and highlighting the challenges of applying these techniques to NTMs.

## One-Step and Two-Step Allelic Exchange Mutagenesis

Allelic exchange mutagenesis harnesses the DNA repair mechanism of homologous recombination (HR) to facilitate replacement of a region of chromosomal DNA. The process utilizes an allelic exchange substrate (AES), which is a piece of DNA that carries the desired mutation and regions of homology to the locus of interest. Following introduction of the AES into the cell, endogenous HR proteins catalyze recombination events such that the homologous region in the chromosome is replaced by the AES (McFadden, 1996). AESs are either suicide vectors, which have no origin of replication, or vectors that replicate under specific conditions, most commonly a specific temperature (Mycobacteria Protocols | SpringerLink) (Parish and Roberts, 2015). For one-step allelic exchange, homologous regions (1,5–2 kb) from either side of the desired deletion are cloned upstream and downstream of an antibiotic resistance marker in a vector backbone to create the AES. A marked deletion is generated when homologous recombination occurs between the AES and the chromosome at both the upstream and downstream regions (double cross-over), i.e., the sequence between the upstream and downstream homologous regions is replaced by the antibiotic resistance marker in the chromosome. Antibiotic selection facilitates isolation of the resulting marked mutants. In two step allelic exchange mutagenesis, AESs contain an additional negative selection marker that results in death of the bacteria during selection, while the antibiotic resistance marker can be positioned in the vector backbone to create unmarked deletions (Parish and Stoker, 2000). The first step of the process uses positive selection (usually antibiotic resistance) to isolate clones that have undergone a single homologous recombination event (single cross-over). This results in integration of the AES, creating a strain that carries both the wild-type and mutant alleles. In the second selection step, the second recombination event is selected for using negative selection, and results in removal of the vector backbone and one of the alleles. Removal of the wild-type allele results in the generation of a mutant strain, and vice versa.

The feasibility of using allelic exchange for mutagenesis in *Mycobacterium intracellulare* was initially demonstrated by showing that homologous recombination resulted in integration of a suicide vector in a site-specific manner (Marklund et al., 1995). One-step allelic exchange mutagenesis was subsequently employed to delete the 19-kilodalton antigen (19Ag) using a suicide vector (Table 1) (Mahenthiralingam et al., 1998). The vector was engineered to contain 6.2 kb of homology, in which the *19Ag* gene had been disrupted by a gentamycin (Gm) cassette. Electroporations performed with 1 μg of the suicide vector and the *M. intracellulare* mouse-avirulent strain, TMC1403, yielded between 8 and 15 Gm^r^ colonies. Only one of these colonies was a marked mutant, indicative of the low efficiency of this method; the remaining colonies were either single cross-overs or had undergone non-homologous recombination. Attempts to use the same suicide vector in the mouse-virulent strain, D673, failed to yield any mutants. In contrast, a mutant derivative of D673, FM1, was amenable to mutagenesis by allelic exchange. The FM1 mutant displayed altered colony morphology, suggesting that it had a cell wall defect that increased its transformation efficiency. This study illustrates that because of low homologous recombination frequencies (10^–4^ to 10^–6^), the low transformation efficiency of NTMs can become a limiting factor in generating mutants.

**TABLE 1:** Examples of one-step and two-step allelic exchange mutagenesis in non-tuberculosis mycobacteria.

Mycobacterial species	DNA delivery method	Target gene(s)	Allelic exchange substrate	Positive selection markers	Negative selection markers	References
**One-step allelic exchange**						
*M. intracellulare* (TMC1403, D673, FM1)	Electroporation	*19Ag*	Suicide vector	Gm^*r*^	n/a	Mahenthiralingam et al. (1998)
*M. avium* subsp. *paratuberculosis* (K10, K10-GFP)	Transduction	*relA pknG lsr2*	Phasmid derived from pHAE87	Km^*r*^ Hyg^*r*^	n/a	Park et al. (2008)
*M. avium* subsp. *paratuberculosis* (K10)	Transduction	*mce4*	Phasmid derived from pHAE87	Km^*r*^	n/a	Alonso et al. (2020)
*M. abscessus* subsp. *abscessus* (CIP104526)	Electroporation	*mmpL4a mmpS4*	Suicide vector	Km^*r*^ mWasabi tdTomato	n/a	Viljoen et al. (2018)
*M. avium* (JCM34) (Leucine auxotroph)	Transduction	*pcaA*	Phasmid derived from pHAE87	Hyg^*r*^ *leuD*	n/a	Otero et al. (2003)
**Two-step allelic exchange**						
*M. avium* (920A6)	Electroporation	*rtfA*	Conditionally replicating vector (ts-*oriM*)	Hyg^*r*^ *xylE*	*sacB*	Irani et al. (2004)
*M. avium* (104)	Electroporation	*mtfD*	Conditionally replicating vector (ts-*oriM*)	Km^*r*^	*sacB*	Krzywinska et al. (2005)
*M. chelonae* (ATCC 35752)	Electroporation	*MCH_4689c MCH_4690c MCH_4691c*	Conditionally replicating vector (ts-*oriM*)	Km^*r*^ or Zeo^*r*^ *xylE*	*sacB*	Moura et al. (2014)
*M. marinum*	Electroporation	*esxB-esxA*	Suicide vector	Hyg^*r*^ *lacZ*	*sacB*	Zhang et al. (2016)
*M. abscessus* (ATCC19977)	Electroporation	*MAB_4395 [aac(2’)]*	Suicide vector	Apr^*r*^	*katG*	Rominski et al. (2017)
*M. abscessus* (ATCC19977)	Electroporation	*mmpL4b mbtH MAB_2875 MAB_2833*	Suicide vector	Km^*r*^ *lacZ*	*galK*	Gregoire et al. (2017)

One strategy to overcome the low transformation efficiency of electroporation is to use mycobacteriophages to deliver DNA. This involves incorporation of the AES (including selection/counter selection markers) into a shuttle phasmid. The most commonly used phasmid, phAE87, is a conditionally replicating derivative of phage TM4 which replicates at 30°C but not at 37°C (Bardarov et al., 2002). Following transduction, bacteria that have undergone recombination are isolated by incubation and selection at the non-permissive temperature (37°C). Attempts to use the phAE87 transduction protocol developed for *Mycobacterium bovis*/*M. tuberculosis* in *Mycobacterium avium* subsp. *paratuberculosis* resulted in a high number of spontaneous mutants and very few allelic exchange mutants (Park et al., 2008). A significantly higher proportion of allelic exchange mutants [78–100% of hygromycin (Hyg) resistant colonies] was obtained using a modified method, modifications that included removal of bacterial clumps prior to transduction and performing selection on a higher antibiotic concentration. Transduction and selection procedures therefore need to be optimized for each NTM species.

In mycobacteria, spontaneous resistance to antibiotics used for selection arises at a similar frequency to homologous recombination (Parish et al., 1999; Bardarov et al., 2002; Viljoen et al., 2018). This can be problematic during allelic exchange mutagenesis as it increases the number of colonies that need to be screened following selection. The emergence of spontaneous mutants is determined by a number of factors, including the number of changes that lead to the phenotype and the resulting fitness cost (Martinez and Baquero, 2000). Using a combination of selection markers or individual markers that develop spontaneous mutants at a lower frequency is therefore a means of overcoming this problem. A system developed for use in *Mycobacteroides abscessus* combined antibiotic selection with fluorescent selection by incorporating tdTomato or mWasabi in the suicide vector (Viljoen et al., 2018). This approach relies on one-step allelic exchange and integration of the suicide vector (single-cross over) into the target gene to disrupt its function. Screening of fluorescent colonies following selection revealed that homologous recombination had occurred in all cases, suggesting that the frequency of non-homologous (illegitimate) recombination in *M. abscessus* is lower than for *M. smegmatis*, *M. bovis* and *M. tuberculosis*. The length of the homology in the suicide vector impacted the frequency of recombination in *M. abscessus*, with regions below 500 bp yielding very few colonies. In *M. avium*, combining antibiotic selection with the *xylE* gene (E.C. 1.13.1.2) enabled identification of recombinant colonies by their yellow appearance in the presence of a catechol solution (Irani et al., 2004). Only 25–40% of the hygromycin colonies stained yellow, indicating a high rate of spontaneous hygromycin resistance in *M. avium.* An alternate strategy employed in *M. avium* was to use the *Streptomyces coelicolor leuD* gene as a selection marker to complement pre-existing leucine auxotrophy (Otero et al., 2003). This marker was chosen because reversion of leucine auxotrophy occurs at a very low frequency (10^–11^). Four of the seven colonies isolated on minimal media had undergone homologous recombination, while non-homologous recombination had occurred in the remaining three colonies. Although this system overcomes the problem of spontaneous mutations, the need for a pre-existing auxotrophic strain limits its application.

The development of a two-step allelic exchange system by Parish and Stoker (2000) has significantly improved the efficiency with which mutations can be introduced in mycobacteria (Parish and Stoker, 2000). This system relies on both antibiotic resistance and β-galactosidase activity (*lacZ*) for positive selection of the first cross-over event, and sucrose sensitivity (*sacB*) for negative selection of the second cross-over event. Two-step allelic exchange using sucrose sensitivity as a counter selection marker has been used to generate mutants in several NTMs, including *Mycobacterium marinum* (Zhang et al., 2016), *M. avium* (Irani et al., 2004; Krzywinska et al., 2005) and *Mycobacteroides chelonae* (Moura et al., 2014) (see Table 1 for further details). The *sacB* marker does not work in *M. abscessus* (Medjahed and Reyrat, 2009), and therefore alternative counter-selection markers have been developed for this organism. The *M. tuberculosis katG* gene encodes the catalase-peroxidase responsible for converting isoniazid into an active form (Vilchèze and Jacobs, 2014). Inclusion of *katG* on two-step AESs sensitizes *M. abscessus* transformants to isoniazid, facilitating counter-selection on isoniazid (Rominski et al., 2017). Similarly, inclusion of the *E. coli galK* gene, which converts 2-deoxygalactose (2-DOG) to the toxic product 2-deoxygalactose-1-phosphate, enables counter selection on 2-DOG (Gregoire et al., 2017). Although two-step allelic exchange is more efficient, the process is slow and several months are required to generate mutants in slow-growing mycobacteria (defined as those that take >7 days to form colonies on a plate).

## Recombineering

**FIGURE 1:**
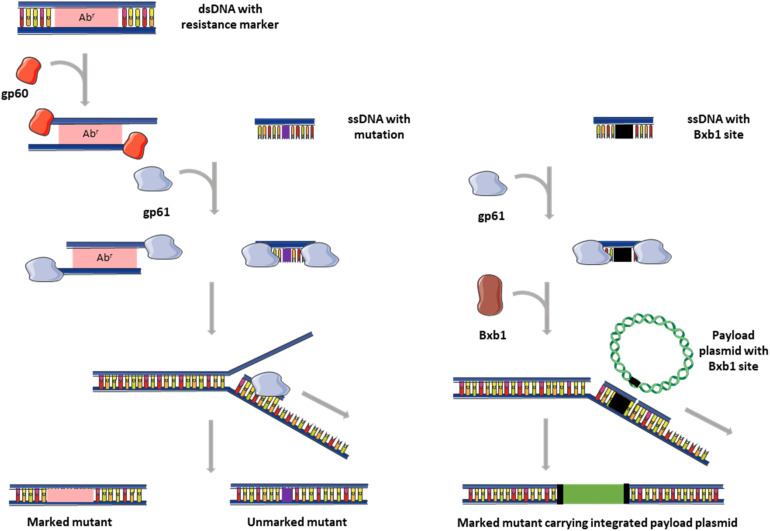
Graphical representation of recombineering with ds and ssDNA and ORBIT. dsDNA substrates (left) usually contain an antibiotic resistance marker for selection, while ssDNA substrates (middle) carry only the desired mutation flanked by short regions (25–50 bp) of homology. In ORBIT (right), the ssDNA substrate contains a Bxb1 integration site flanked by short regions (25–50 bp) of homology. The gp60 exonuclease (RecE homolog) generates single stranded overhangs from the dsDNA fragments, while gp61 (RecT homolog) promotes annealing of ssDNA to homologous regions in the chromosome. Recombineering is more efficient when oligonucleotides are targeted to the lagging strand (indicated in diagram). In ORBIT, Bxb1 facilitates concomitant integration of the payload plasmid. Images adapted from Servier Medical Art by Servier which is licensed under a Creative Commons Attribution 3.0 Unported License (https://smart.servier.com/).

Recombineering is a homologous recombination process mediated by the expression bacteriophage-encoded recombination enzymes in bacterial cells. In this method, the AESs are linear DNA fragments, which are either double-stranded (dsDNA) or single-stranded (ssDNA), depending on the phage proteins being used (Murphy, 1998). Two recombineering systems are commonly used in *E. coli* namely, the lamda (λ) Red system, comprised of three proteins, Exo, Beta and Gam (Yu et al., 2000), and the Rac prophage system, which requires RecE and RecT proteins (Zhang et al., 1998). The Exo and RecE exonucleases function to generate a ssDNA tail or overhangs from a linear dsDNA, while the RecT and Beta proteins bind to ssDNA promoting annealing to the homologous chromosomal region (Figure 1). The λ Red Gam protein inhibits the host’s recombination system to prevent dsDNA degradation. Expression of phage-encoded recombineering proteins in *E. coli* increases recombination efficiency between 10- and 100-fold (Zhang et al., 1998; Yu et al., 2000).

The λ Red and Rac systems do not function efficiently in mycobacteria, and therefore homologs from the mycobacteriophage Che9c, gp60 (RecE homolog) and gp61 (RecT homolog), were investigated for their ability to mediate recombineering (van Kessel and Hatfull, 2007; van Kessel et al., 2008). Recombineering with a dsDNA substrate in *M. smegmatis* revealed that while efficiency was dependent on the length of homology (increased efficiency with AESs between 50 bp and 500 bp), the absolute number of transformants recovered was limited by the competence of the cells. An AES substrate containing 500 bp of homology on either side of a hygromycin gene yielded recombination frequencies of between 2.4 × 10^–5^ and 2.8 × 10^–4^, with >90% of the colonies being recombinants (van Kessel and Hatfull, 2007). Similar frequencies were observed in the slow-grower, *M. tuberculosis*. The inclusion of *res*, FRT or *loxP* sites on either sides of the antibiotic resistance gene in the dsDNA AES facilitates removal of the marker after mutant selection (van Kessel and Hatfull, 2007; Kenan, 2015). Another improvement to the system was the use of plasmids with ts-*oriM*s and counter-selection markers (*sacB*) to facilitate curing of the gp60/gp61-expressing plasmid after recombineering, thereby preventing subsequent chromosomal rearrangements (van Kessel et al., 2008). Recombineering using short ssDNA (50–100 bp) is considerably more efficient than dsDNA recombineering (10- to 100-fold), although this is also limited by cell competence. Single stranded AESs that target the lagging strand are more efficient, with the degree of this bias depending on chromosomal location; a 10,000-fold difference has been observed in some loci (van Kessel et al., 2008). Unlike dsDNA recombineering, ssDNA recombineering in slow growing mycobacteria is 5- to 10-fold less efficient than *M. smegmatis* (van Kessel and Hatfull, 2008). Since these short AESs do not carry a selection marker, identification of mutants can require screening of up to a 1000 single clones in the absence of a selectable phenotype, which is a major drawback of this technique (Kenan, 2015).

Currently, very few reports of recombineering in NTMs exist in the literature. Double stranded recombineering was successfully used in *M. chelonae* (ATCC 35752) to disrupt three porin genes (*MCH_4689c, MCH_4690c*, and *MCH_4691c*) using a plasmid expressing gp60 and gp61 (Moura et al., 2014). Electroporation of the ATCC 35752 strain with 300 ng of each AES yielded between 29 and 65 Zeo^r^ colonies, and between 30 and 80% of these colonies had undergone allelic exchange. Interestingly, the plasmid expressing the recombineering proteins was rapidly lost from *M. chelonae* in the absence of selection (1–2 passages), and therefore counter-selection or the use of conditionally replicating plasmids was not required. Use of dsDNA recombineering to disrupt *mmpL4b* in *M. abscessus* (CIP104536T) was significantly less efficient, with only 7% of clones undergoing allelic exchange. The reason for the low efficiency in *M. abscessus* is unclear and suggests that the utility of the method may be species-specific. To date, the only report of ssDNA recombineering in NTMs is in *M. marinum*, where it was used in combination with CRISPR/Cas12a, and is discussed below.

## Oligonucleotide-Mediated Recombineering Followed by Bxb1 Integrase Targeting (ORBIT)

Oligonucleotide-mediated recombineering followed by Bxb1 integrase targeting (ORBIT) was developed by combining of two efficient recombination systems, namely homologous recombination and site-specific integration (Murphy et al., 2018). The technique is mediated by the co-expression of the gp61 annealase and site-specific Bxb1 integrase, and involves co-transformation of a ssDNA targeting oligonucleotide (containing a Bxb1 recombination site) and a payload plasmid (containing an antibiotic selectable marker and a Bxb1 recombination site) (Figure 1). The gp61 annealase mediates recombineering of the oligonucleotide to introduce a Bxb1 site in a site-specific manner, while Bxb1 facilitates concomitant integration of the payload plasmid (Murphy et al., 2018). A library of payload plasmids was generated to facilitate use of the technique for promoter replacements, deletions and the introduction of C-terminal fusions. Application of ORBIT in *M. smegmatis* and *M. tuberculosis* yielded 20–200 clones for each transformation (using 1 μg oligonucleotide and 200 ng payload plasmid). In ORBIT, the Bxb1 integration system is independent of host factors because both the integrase and recombination sites are provided. The feasibility of using ORBIT in NTM species would therefore depend on the efficiency of the gp61 to mediate recombineering.

## Random Mutagenesis Using Transposons

Transposons are mobile genetic elements that in addition to the genes essential for transposition, also have genes encoding a phenotypic trait, such as antibiotic resistance. Two types of transposition events exist, namely replicative and cut-and-paste (conservative) transposition (Griffiths et al., 2000). In replicative transposition co-integration between the transposon delivery replicon and the target DNA occurs in such a way that transposon is duplicated. A site-specific recombinase subsequently resolves the co-integrant to reform the delivery replicon, leaving a single copy of the transposon in the target DNA. In cut-and-paste transposition, a transposase binds to a transposon creating a synaptic complex, cleaving it from the donor molecule and inserting it into the target site without duplication. Cut-and-paste transposition is typical for Tn*5*, Tn*10* and mariner transposons.

The first insertional mutant libraries in *M. smegmatis* (*mc^2^155)* were created using the Tn*611* transposon, a derivative of the *Mycolicibacterium fortuitum* transposon Tn*610* (Martin et al., 1990). Initially the transposon was used with non-replicating plasmids for mutagenesis, but this resulted in low transposition efficiency. Combining Tn*611* with a conditionally replicating plasmid (ts-*oriM*) improved efficiency by facilitating initial selection of transformants at 30°C and subsequent selection of mutants at 39°C (non-permissive temperature). Transposition of Tn*611* occurs by the replicative mechanism, and in *M. smegmatis*, no insertion hotspots were observed (Guilhot et al., 1994). Culturing of transposon mutants without antibiotic selection revealed a low reversion frequency, indicating that Tn*611* stabilizes upon host chromosome integration (Guilhot et al., 1994). The transposons Tn*5367*, Tn*5368* and Tn*5370* are derived from the *M. smegmatis* IS*1096* insertion sequence and differ from Tn*611* in that the mechanism of transposition is cut-and-paste (McAdam et al., 1995). Transposon insertion is random, and analysis of the insertion sites identified a weak consensus sequence for insertion (5′-NNP y(A/T)A(A/T)NN-3′), showing a preference for an AT-rich center.

As for allelic exchange, temperature sensitive phages, such as phAE77 and phAE94, have been used for more efficient transposon delivery. In *M. marinum*, transduction with phAE94 carrying Tn*5367* yielded 10^5^ Km^r^ colonies per ml of the transduction mixture. This was significantly more efficient than the phAE77 derivative which yielded only 10^3^ per ml (Rybniker et al., 2003), demonstrating that phage choice influences efficiency (Table 2). Transposition efficiency is also influenced by experimental conditions. For example, in *M. avium subsp. paratuberculosis (K-10)* strain optimal transposition frequencies (10^–6^ to 10^–7^ per recipient cell) were obtained after 4 h of co-incubation of bacteria and phages, while the frequency dropped 10-fold with longer incubation times (Harris et al., 1999). Tn*5367* mutagenesis in both *M. marinum* and *M. avium subsp. paratuberculosis (K-10)* revealed that insertions were flanked with 8 bp target duplication, and in *M. marinum* insertion of small parts of the phage DNA was observed in some mutants (Harris et al., 1999; Rybniker et al., 2003). Use of the phAE94/Tn*5367* system in *Mycobacterium ulcerans* resulted in a high number of mutants with slow growth, limiting DNA isolation for Southern blot hybridization (Rybniker et al., 2003). Although PCR screening suggested transposon insertion, plaque formation at 32°C led the authors to speculate that integration of the prophage had occurred. phAE94 may therefore be functioning as a temperate phage in *M. ulcerans*, highlighting the need to confirm temperature-sensitive replication in different species.

**TABLE 2:** Examples of random transposon mutagenesis in non-tuberculosis mycobacteria (NTMs).

Mycobacterial species	Transposon	Plasmid/phage	Selection marker(s)	Purpose/selection	References
*M. marinum*	IS*1096* IS*6110*	pYUB285 pUS252 (suicide plasmids)	Km^r^	Insertional library	Talaat and Trucksis (2000)
*M. marinum*	Tn*5367*	phAE94 (TM4) phAE77 (D29) (ts phasmids)	Km^r^	Insertional library	Rybniker et al. (2003)
*M. marinum*	Mos1	pM272B (ts-*oriM*)	Km^r^ *sacB*	Insertional library, pigmentation mutants	Gao et al. (2003)
*M. marinum*	MycoMarT7	phAE94 (TM4)	Km^r^	Essentiality screen	Weerdenburg et al. (2015)
*M. avium* subsp. *paratuberculosis*	Tn*5367*	phAE94 (TM4)	Km^r^	Insertional library	Harris et al. (1999)
*M. avium* subsp. *paratuberculosis*	Tn*5367* MycoMarT7	phAE94 (TM4) phAE94 (TM4)	Km^r^	Insertional library	Rathnaiah et al. (2016)
*M. avium*	MycoMarT7	phAE94 (TM4)	Km^r^	Insertional library, antibiotic susceptibility	Cangelosi et al. (2006)
*M. avium* subsp. *hominissuis*	MycoMarT7	phAE94 (TM4)	Km^r^	Essentiality screen	Dragset et al. (2019)
*M. avium* subsp. *avium* (HMC02)	EZ::TN	n/a	Km^r^	Insertional library Ciprofloxacin^*s*^	Cangelosi et al. (2006)
*M. kansasii*	MycoMarT7	phAE94 (TM4)	Km^r^	Insertional library, colony morphology	Budell et al. (2020)
*M. intracellulare*	MycoMarT7	phAE94 (TM4)	Km^r^	Essentiality screen	Tateishi et al. (2020)
*M. fortuitum*	Tn*phoA*	pRT291 (suicide vector)	Km^r^ *phoA*	Library with insertions in secreted/membrane proteins, resistance to acid stress biofilm defect	Poonam et al. (2019); Katoch et al. (2020)
*M. ulcerans*	Tn*5367*	phAE94 (TM4)	Km^r^	Unsuccessful	Rybniker et al. (2003)

Mariner transposons have recently been developed as genetic tools for use in bacteria and the most commonly used are *Mos1* and *Himar1*. One of the major advantages of the mariner transposons are their reduced recognition sequence (5′-TA-3′), which is less restrictive. The first mariner transposon used for mutagenesis in *M. marinum* was *Mos1* isolated from *Drosophila melanogaster*. A delivery vector, pM272B, was engineered to contain the transposon with a kanamycin resistance gene, a thermosensitive origin of replication and a *sacB* gene used for counter selection (Gao et al., 2003). Following initial selection of kanamycin (Km) resistant transformants after electroporation, transposition was observed at a rate of approximately 3 × 10^2^ Km resistant and sucrose sensitive bacteria per 10^5^ Km resistant bacteria. Analysis of the transposon junctions revealed a TA dinucleotide insertion site followed by GC-rich genomic sequences. The mariner transposon inserted randomly, usually with one transposon copy per genome, and the delivery vector was lost upon transposition.

The most-frequently used transposon in mycobacterial research is the *Himar-1*-derived MycoMarT7 mariner transposon. This transposon has been engineered to contain a T7 promoter, used for insertion site identification, and a kanamycin selection marker. It is usually delivered using a temperature-sensitive phage. A comparison of Tn*5367* and the MycoMarT7 mariner transposon in *M. avium subsp. paratuberculosis* revealed a >3-fold higher number of insertions for MycoMarT7, and a higher percentage of insertions occurring within genes (83% vs. 74%) (Rathnaiah et al., 2016). The MycoMarT7 transposon is therefore superior at creating a comprehensive library. Despite the reduced recognition sequence, transposition bias was detected for MycoMarT7, and it was estimated that 400,000 *M. avium subsp. paratuberculosis* mutants would be required for a representative library. This is four times higher than the estimate for *M. tuberculosis* and such biases could result in an overestimation of the number of essential genes in forward genetic screens in this organism. Evaluation of ΦMycoMarT7 in *Mycobacterium kansasii* revealed that while temperature sensitivity was maintained, plaque formation in *M. kansasii* was 800-fold less efficient than for *M. smegmatis* (Budell et al., 2020). Mapping of the insertion sites for the 14,700 *M. kansasii* mutants revealed that 82% of the insertions in the chromosome were in open reading frames (ORFs); a similar percentage (83%) was observed for the pMK12478 plasmid present in this strain. The slightly higher percentage of insertions in annotated genes in the plasmid (74% vs. 62%) could be due to the non-essential nature of genes present extra-chromosomally, however, since the library is not saturated the data is not conclusive.

Most studies in NTMs have used transposon mutagenesis to select for mutants with specific phenotypes. Examples include pigmentation variants in *M. marinum* (Gao et al., 2003), *M. kansasii* mutants with altered colony morphology (Budell et al., 2020) and *M. avium* mutants with altered antibiotic susceptibility (Cangelosi et al., 2006) (see Table 2 for details). The Tn*phoA* transposon was developed to perform more targeted screens that identify mutants with insertions in membrane or secreted proteins (Taylor et al., 1987). The Tn*phoA* transposon encodes an alkaline phosphatase enzyme, but the gene is lacking its signal sequence (Katoch et al., 2020). If the transposon inserts into a gene encoding a membrane or secreted protein, the presence of alkaline phosphatase at the cell surface turns the colony blue when selected on 5-bromo-4-chloro-3-indolyl phosphate (XP). A Tn*phoA* transposon mutant library in *M. fortuitum* yielded 42 mutants with alkaline phosphatase activity, approximately 9% of the total number of mutants isolated (Katoch et al., 2020). Analysis of a mutant (MT721) with a biofilm formation defect identified a deletion in the membrane protein anthranilate phosphoribosyl transferase (TrpD), confirming the utility of this approach (Katoch et al., 2020).

One of the limitations of using transposons for mutagenesis is that a functional transposase must be produced within the host. The use of transposomes, which is a stable complex between a purified transposase enzyme and the transposon, overcomes this limitation (Goryshin et al., 2000). A commercial transposome system derived from Tn*903*, EZ-Tn (Epicenter), has been developed for use in a broad host range, and was used to generate a mutant library of 3500 mutants in the *M. avium* subsp. *avium HMC02* (Laurent et al., 2003). The efficiency of transposition efficiency of EZ-Tn in certain *M. avium* morphotypes was significantly lower, presumably due to transposome delivery being influenced by cell wall composition. Low transformation efficiency is therefore a limitation of this system.

In addition to generating mutants, transposon mutagenesis can be used for the genome-wide prediction of essential genes. This involves the generation of saturating transposon mutant libraries (hundreds of thousands of mutants), and the mapping of the transposon insertion site in every mutant in the library. By comparing the frequency of observed insertions to all possible insertion sites within a gene, essential genes can be identified. In addition, genes that confer a growth advantage or defect can be identified by comparing the abundance of mutants in the library. This approach has been used with the MycoMarT7 transposon to identify essential genes in *M. marinum* (Weerdenburg et al., 2015), *Mycobacterium avium* subsp. *hominissuis* (Dragset et al., 2019) and *Mycobacterium intracellulare* (Tateishi et al., 2020) (see Table 2 for details).

## Random Mutagenesis by Non-Homologous Recombination

Although high levels non-homologous recombination is undesirable when introducing site-specific mutations, its non-specific nature can be harnessed to introduce random mutations. The high levels of non-homologous recombination in slow-growing mycobacteria has been exploited to generate mutants in *M. bovis* (Wilson et al., 1997) and *M. avium* (Khattak et al., 2012). The study in *M. bovis* was initially aimed at investigating the role of the *aphC* gene, and therefore used an AES containing a kanamycin resistance gene (*neo*) flanked by sequences from the *M. bovis aphC* gene. Of the 440 transformants containing an insertion, none disrupted the *aphC* gene, indicating that non-homologous rather than homologous recombination had occurred (Wilson et al., 1997). Since the flanking regions did not appear to confer any specificity, the study in *M. avium* subsp. *hominissuis* used a linear DNA fragment containing a hygromycin resistance gene flanked by vector (pYUB854) sequences. Electroporation of 3–6 μg of this linear DNA fragment yielded about 1000 Hyg^r^ colonies (Khattak et al., 2012). Analysis of 13 randomly chosen mutants revealed that the resulting deletions ranged between 2 and 669 bp, and 12 of the 13 deletions had disrupted a single gene. While this method is easier to perform than transposon mutagenesis, it is significantly less efficient.

## Mutagenesis Using CRISPR/Cas

**FIGURE 2:**
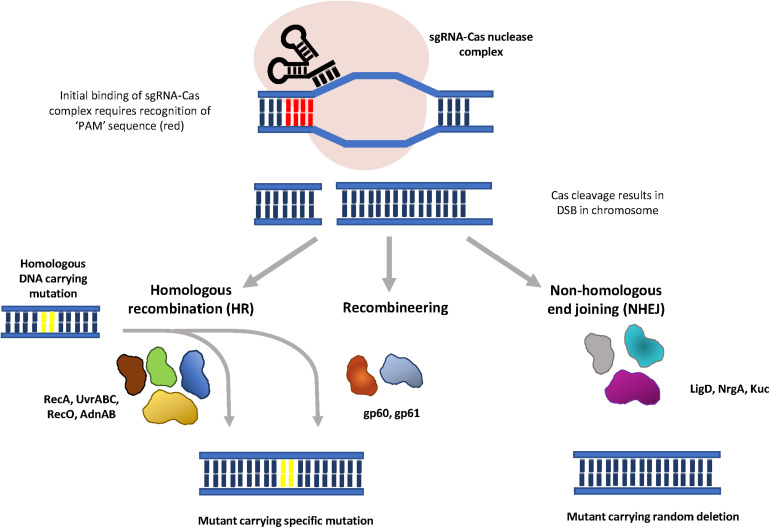
Graphical representation of CRISPR/Cas mutagenesis. Synthetic sgRNAs (black) direct binding of the Cas nuclease (pink) to a specific gene through Watson-Crick base paring between the sgRNA and the target sequence. Initial binding of the sgRNA-Cas complex is dependent on the recognition of an adjacent PAM sequence (red). Nuclease cleavage results in a double strand break in the chromosome, which is repaired either through homologous recombination, in the presence of a homologous substrate, or non-homologous end joining. The error-prone nature of NHEJ it results in random mutations at the site of repair. Phage proteins gp60 and gp61 are proposed to function in DSB repair with ssDNA and dsDNA substrates (CRISPR/Cas-mediated recombineering).

The Clustered Regularly Interspaced Palindromic Repeats (CRISPR)/CRISPR associated protein (Cas) systems in prokaryotes act as an adaptive immune system which renders bacteria resistant to repeated phage infection (Marraffini, 2015). Following infection, phage DNA is digested by the host and selected fragments are integrated into the CRISPR locus as short sequences, termed spacers (Bolotin et al., 2005; Mojica et al., 2005; Pourcel et al., 2005). The integrated DNA is transcribed to produce a mature transcript, crRNA, which will direct the Cas nuclease to cleave sequences that are complementary to the crRNA in the phage genome during subsequent infections. The specificity and programmability of the CRISPR/Cas system makes it an attractive tool for mutagenesis, and the CRISPR/Cas9 and CRISPR/Cas12a systems have been widely utilized to introduce mutations in a range of organisms. By using synthetic small guide RNAs (sgRNA) to mimic crRNA, a Cas-sgRNA complex can be directed to a specific gene by Watson-Crick base pairing between the sgRNA and the target DNA sequence (Figure 2). The Cas nuclease then produces a double strand break (DSB) in the target gene, which must be repaired by the host to maintain chromosomal integrity. The DSB repair is performed either by homologous recombination (HR), using a corresponding homologous template, or by non-homologous end joining (NHEJ), in absence of a homologous template (Featherstone and Jackson, 1999). Because NHEJ is prone to insertions and deletions, this can lead to mutations in the target gene.

The first reported use of the CRISPR/Cas9 system in mycobacteria was for gene silencing by CRISPR interference (CRISPRi) (Choudhary et al., 2015). Genes can be silenced by mutating the endonuclease domain of the Cas9 to make it catalytically inactive (dCas9) (Larson et al., 2013). The sgRNA-dCas9 complex binds to the target DNA sequence, however, since dCas9 cannot cleave the DNA, the complex remains bound, blocking the binding or movement of RNA polymerase. This results in gene-specific transcriptional repression by blocking either initiation or elongation, depending on the position of the sgRNA-dCas9 complex relative to the start of the gene (Qi et al., 2013). Evaluation of 11 Cas9 proteins in mycobacteria revealed that the enzyme from *Streptococcus thermophilus* (dCas9_Sth1_) was most efficacious in the knock-down of endogenous gene expression (Rock et al., 2017). CRISPRi is a major advance in studying gene function due to its simplicity. The technique has one major drawback in that when genes are operonic, all genes downstream of the target gene are also silenced.

In most DNA-targeting CRISPR-Cas systems, initial binding requires recognition of a protospacer adjacent motif (PAM), a short (3 to 5 bp) DNA sequence immediately upstream of the sequence recognized by the crRNA (Marraffini, 2015). Since Cas9 and Cas12a nucleases recognize different PAMs (Leenay et al., 2016), using different Cas nucleases facilitates targeting of different sites in the chromosome. The CRISPR/Cas12a system was first used in *M. smegmatis* to increase recombineering efficiency (Yan et al., 2017). A two-plasmid system, (also demonstrated to function in *E. coli* and *Yersinia pestis*) coupled an Cas12a endonuclease from *Francisella novicida* (*Fn*Cpf1) with the recombineering proteins gp60 and gp61 (Figure 2), to create markerless, scarless mutations. Recombineering using ssDNA was 50-times more efficient when combined with *Fn*Cpf1-site specific cleavage. The expected strand bias was observed, with efficiencies of 80% and 69% obtained for oligonucleotides targeted to the lagging strand, while the leading-strand oligonucleotide had a fivefold lower efficiency. The efficiency of introducing insertions and deletions by CRISPR-FnCpf1-enhanced recombineering is size – dependent. For insertions, 27% of transformants had 5 bp insertions, 10% had 10 bp insertions, while only 3.1% had 20 bp insertions (Yan et al., 2017). Similarly, the efficiency for introducing small deletions (5, 10, 20 bp) ranged between 70% and 90%, while significantly lower efficiencies of 17.4% and 8.2% were observed for 418 bp and 1000 bp deletions, respectively. In addition, CRISPR-*Fn*Cpf1-enhanced recombineering using 1 kb dsDNA fragments facilitated the introduction of markerless deletions of up to 4000 kb with an efficiency of greater than 45% (Yan et al., 2017). The limitation of the Yan et al. (2020) study was that the requirement for gp60 and gp61 was not demonstrated, and therefore the role of endogenous homologous recombination proteins in the process is not clear. The major advantage of CRISPR/Cas-enhanced recombineering is that it takes approximately half the time of two-step allelic exchange, however, since it did not work in *M. tuberculosis*, it may not function in all NTMs (Yan et al., 2020).

The *Fn*Cpf1 nuclease was unable to generate mutants in *M. smegmatis* in the absence of recombineering substrates, suggesting that NHEJ was not functioning to repair DSB generated by the nuclease (Sun et al., 2018). In contrast, expression of the *Fn*Cpf1 nuclease and a crRNA targeting a non-essential gene in *M. marinum* resulted in deletions ranging from 2 bp to 10 179 bp (Yan et al., 2020). Deletion of *ku* and *ligD* genes in *M. marinum* reduced genome editing efficiency to below 10%, while complementation increased NHEJ editing efficiency to 90%. Furthermore, expression of the *M. marinum* NHEJ (MmNHEJ) locus with the CRISPR-FnCpf1 system in *M. smegmatis* resulted mutations in 0.75% of transformants. This efficiency was increased to 90% when the experiment was performed in a *recA* null mutant, suggesting that inhibiting HR is required for the NHEJ system to repair DSBs in *M. smegmatis*.

To facilitate efficient CRISPR-*Fn*Cpf1-assisted NHEJ genome editing in mycobacteria a two-plasmid system was subsequently developed; one plasmid expresses RecA_mu_ (dominant RecA negative mutant), RecX (RecA regulator), *Mm*NHEJ (*M. marinum* NHEJ locus), while the other expresses *Fn*Cpf1 and the crRNA (Yan et al., 2020). These promoted the NHEJ genome editing in *M. smegmatis* with high efficiency (80–90%). Replacement of the *Fn*Cpf1 with Cas9_Sth1_ (Cas9 from *Streptococcus thermophilus*) achieved genome editing efficiencies of above 80% in both *M. smegmatis* and *M. tuberculosis*, and deletions of between 1 and 324 bp observed at the cleavage site in *M. tuberculosis*. The method may therefore have some utility for introducing random mutations in other mycobacterial species.

## Concluding Remarks

Despite advances in mycobacterial mutagenesis, a limiting factor for all techniques is the efficient delivery of DNA into cells. Due to the complex nature of the mycobacterial cell wall, electroporation efficiency is low in many species. The *M. smegmatis* mc^2^155 strain, which has become a workhorse in mycobacterial research, is a mutant that was isolated for its high transformation efficiency, an efficiency 10^5^ times greater than its parental strain (Snapper et al., 1990). Transformation efficiency differs both between mycobacterial species and between strains within a species. For example, strains of *M. avium* display transformation efficiencies between 10^2^ and 10^4^ (Foley-Thomas et al., 1995; Lee et al., 2002), while efficiencies of 10^5^ have been reported for *M. bovis* BCG and *M. tuberculosis* H37Rv (Wards and Collins, 1996). Although optimization of the electroporation procedure may afford some improvement (Wards and Collins, 1996; Lee et al., 2002), in some species the isolation of transformation efficient mutants may be required (Snapper et al., 1990; Mahenthiralingam et al., 1998; Dragset et al., 2019). Delivery of DNA using conditionally replicating mycobacteriophages is a more efficient alternative to electroporation, although this requires additional steps to propagate the phages in a suitable host. Most of the phasmids used in mycobacterial research are derived from phage TM4, which has a broad host range. Differences in transfection efficiencies between species and strains have been observed (Foley-Thomas et al., 1995; Dragset et al., 2019) and optimization of transduction protocols may be required (Park et al., 2008). Another consideration when using temperature sensitive phages, is the functionality of the ts-*oriM* in the mycobacterial species being manipulated (Rybniker et al., 2003).

Differences in the physiology of mycobacterial species can impact on the utility of a mutagenesis technique and should therefore be considered when choosing an approach. Examples here include the functionality of selection markers (such as *sacB* in *M. abscessus*) or the rate of spontaneous antibiotic resistant mutants arising in an organism; a property determined both by the mechanisms of resistance and the DNA repair pathways in that species. The differential functionality of CRISPR-mediated mutagenesis in *M. smegmatis*, *M. tuberculosis* and *M. marinum* highlights how differences in DNA repair mechanisms in different species can influence the choice of technique (Yan et al., 2020). In *M. smegmatis*, CRISPR-*Fn*Cpf1 cleavage could enhance recombineering, however, the system did not work in *M. tuberculosis*. Similarly, the NHEJ pathway could repair DSB introduced by the *Fn*Cpf1 nuclease in *M. marinum*, but not in *M. smegmatis*. Given that studies investigating the physiology of NTMs remain limited, choice of mutagenesis techniques in many species often involves trial and error. A concerted effort to both optimize mutagenesis tools for use in NTMs and to understand the underlying physiology influencing their efficacy, is therefore needed to advance the genetic manipulation of NTMs.

## Author Contributions

NC wrote manuscript. MW wrote and edited manuscript. Both authors contributed to the article and approved the submitted version.

## Conflict of Interest

The authors declare that the research was conducted in the absence of any commercial or financial relationships that could be construed as a potential conflict of interest.
